# {μ-1,2-Bis[bis­(4-meth­oxy­phen­yl)phosphan­yl]-1,2-diethyl­hydrazine-κ^2^ *P*:*P*′}bis­[chloridogold(I)] tetra­hydro­furan disolvate

**DOI:** 10.1107/S1600536811038499

**Published:** 2011-09-30

**Authors:** Frederik H. Kriel, Manuel A. Fernandes, Judy Coates

**Affiliations:** a AuTEK, Mintek, Private Bag X3015, Randburg, 2125, South Africa; b Molecular Science Institute, School of Chemistry, University of the Witwatersrand, PO Wits, 2050, Johannesburg, South Africa

## Abstract

The title compound, [Au_2_Cl_2_(C_32_H_38_N_2_O_4_P_2_)]·2C_4_H_8_O, is formed from a bidentate phosphine ligand complexed to two linear gold(I) nuclei [P—Au—Cl = 175.98 (3)°]. The nuclei are 3.1414 (2) Å apart. The mol­ecule exhibits a twofold symmetry axis. Stacks of the compound are formed through inter­molecular C—H⋯Cl inter­actions, while the tetra­hydro­furan (THF) solvate is further attached to the stacks through weak C—H⋯O hydrogen bonding from the THF O atom to two separate H atoms on the complex.

## Related literature

For the synthesis of the parent ligand and related structures utilizing alternative metals see: Reddy *et al.* (1994 ▶, 1995 ▶); Slawin *et al.* (2002 ▶); Kriel *et al.* (2011*a* ▶,**b* ▶,c* ▶). For Au⋯Au inter­actions, see: Holleman & Wiberg (2001 ▶). For the biological activity of the title complex, see: Fonteh & Meyer (2009 ▶).
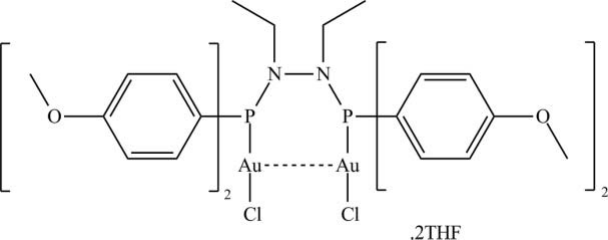


## Experimental

### 

#### Crystal data

[Au_2_Cl_2_(C_32_H_38_N_2_O_4_P_2_)]·2C_4_H_8_O
*M* *_r_* = 1185.63
Monoclinic, 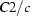

*a* = 23.6375 (4) Å
*b* = 9.1260 (1) Å
*c* = 20.2269 (3) Å
β = 93.976 (1)°
*V* = 4352.76 (11) Å^3^
*Z* = 4
Mo *K*α radiation
μ = 6.98 mm^−1^
*T* = 173 K
0.36 × 0.20 × 0.07 mm

#### Data collection

Bruker APEXII CCD diffractometer
Absorption correction: integration (*SADABS*; Bruker, 1999 ▶) *T*_min_ = 0.188, *T*_max_ = 0.641
38471 measured reflections
5253 independent reflections
4694 reflections with *I* > 2σ(*I*)
*R*_int_ = 0.046

#### Refinement

*R*[*F* ^2^ > 2σ(*F* ^2^)] = 0.022
*wR*(*F* ^2^) = 0.059
*S* = 1.05
5253 reflections
244 parameters
H-atom parameters constrained
Δρ_max_ = 1.74 e Å^−3^
Δρ_min_ = −0.64 e Å^−3^

### 

Data collection: *APEX2* (Bruker, 1999 ▶); cell refinement: *SAINT-Plus* (Bruker, 1999 ▶); data reduction: *SAINT-Plus*; program(s) used to solve structure: *SHELXS97* (Sheldrick, 2008 ▶); program(s) used to refine structure: *SHELXL97* (Sheldrick, 2008 ▶); molecular graphics: *Mercury* (Macrae *et al.*, 2006 ▶); software used to prepare material for publication: *WinGX* (Farrugia, 1999 ▶).

## Supplementary Material

Crystal structure: contains datablock(s) I, New_Global_Publ_Block. DOI: 10.1107/S1600536811038499/bh2379sup1.cif

Structure factors: contains datablock(s) I. DOI: 10.1107/S1600536811038499/bh2379Isup2.hkl

Additional supplementary materials:  crystallographic information; 3D view; checkCIF report

## Figures and Tables

**Table 1:** Hydrogen-bond geometry (Å, °)

*D*—H⋯*A*	*D*—H	H⋯*A*	*D*⋯*A*	*D*—H⋯*A*
C1—H1*A*⋯Cl^i^	0.99	2.80	3.592 (3)	137
C15—H15⋯O3^ii^	0.95	2.63	3.497 (5)	152
C17—H17*B*⋯O3^ii^	0.98	2.53	3.444 (7)	156

Symmetry codes: (i) 
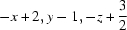
; (ii) 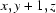.

**Fig. 1.**
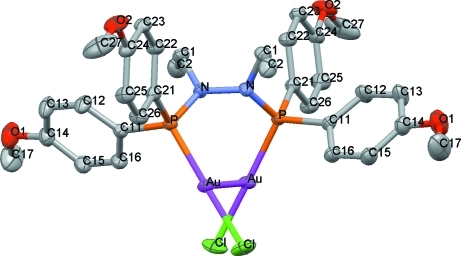
Molecular structure of the title complex drawn with displacement ellipsoids at the 50% probability level. Hydrogen atoms and solvent THF have been omitted for clarity.

**Fig. 2.**
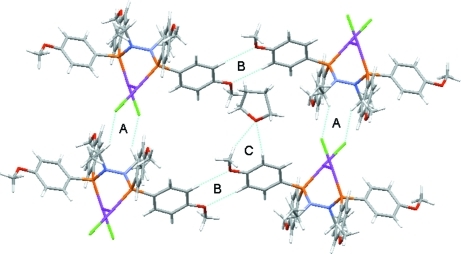
Packing of the title compound showing short contacts (dashed lines).

## supplementary crystallographic information

### Comment

The title compound, C_32_H_38_Au_2_Cl_2_N_2_O_4_P_2_.2(C_4_H_8_O), formed from a bidentate phosphine ligand complexed to two linear gold(I) nuclei, readily crystallizes out of dichloromethane (DCM) with the addition of a few drops of tetrahydrofuran (THF). The crystal structure includes two THF solvent molecules. The complex molecule is bisected by a twofold axis through the N—N' and Au—Au' lines (Fig. 1). Gold(I) has an almost linear coordination with a P—Au—Cl angle of 175.98 (3)°. The Au—Au distance within the complex is 3.141 (2) Å, well within the range of aurophilic interactions (described in Holleman & Wiberg, 2001, as being normally between 2.7 Å and 3.4 Å). Other bond lengths are within expected ranges.

The structure exhibits columns of complexes arranged head-to-tail along *b*, forming channels filled with THF. There is an intracolumnar contact involving Cl atoms in one molecule and H atoms on the ethyl substituted hydrazine bridge of a neighboring one, in the same column (Cl···H1A*^i^*: 2.80 Å, (*i*): 2-*x*, -1+*y*, 3/2-*z*; site A in Fig. 2). There are also weak intercolumnar H-bonding contacts (O1···H13*^ii^*: 2.61 Å, (*ii*): 3/2-*x*, 1/2-*y*, 1-*z;* site B in Fig. 2). Finally, the THF solvate molecule is weakly attached to the columns by a pair of O···H contacts (O3···H15 = 2.63 Å, O3···H17B = 2.53 Å; site C in Fig. 2).

The biological activity of the title complex is discussed in Fonteh & Meyer (2009), and related structures with other dialkyl hydrazine derivatives have been also reported (Kriel *et al.*, 2011*a*,*b*,*c*; Reddy *et al.*, 1994, 1995; Slawin *et al.*, 2002).

### Experimental

The complex was synthesized by dissolving tetrahydrothiophenegold(I) chloride [(THT)AuCl] in DCM and adding 0.5 equivalents of the corresponding ligand, bis(di(4-methoxyphenyl)phosphino)-1,2-diethylhydrazine. The addition of a few drops of THF led to the growth of crystals suitable for use in single-crystal X-Ray analysis. The presence of THF during the initial complexation led to undesirable side products as a result of the breakdown of the ligand.

### Refinement

The H atoms were positioned geometrically and allowed to ride on their respective parent atoms, with C—H = 0.95 (aromatic CH), 0.99 (CH_2_) or 0.98 (CH_3_) Å, and with *U*_eq_ = 1.2 (CH, CH_2_) or 1.5 (CH_3_) times *U*_eq_(C).

### Crystal data

**Table:**

[Au_2_Cl_2_(C_32_H_38_N_2_O_4_P_2_)]·2C_4_H_8_O	*F*(000) = 2312
*M**_r_* = 1185.63	*D*_x_ = 1.809 Mg m^−^^3^
Monoclinic, *C*2/*c*	Melting point: 369 K
Hall symbol: -C 2yc	Mo *K*α radiation, λ = 0.71073 Å
*a* = 23.6375 (4) Å	Cell parameters from 7364 reflections
*b* = 9.1260 (1) Å	θ = 2.4–34.2°
*c* = 20.2269 (3) Å	µ = 6.98 mm^−^^1^
β = 93.976 (1)°	*T* = 173 K
*V* = 4352.76 (11) Å^3^	Plate, colourless
*Z* = 4	0.36 × 0.20 × 0.07 mm

### Data collection

**Table:**

Bruker APEXII CCD diffractometer	5253 independent reflections
Radiation source: fine-focus sealed tube	4694 reflections with *I* > 2σ(*I*)
graphite	*R*_int_ = 0.046
φ and ω scans	θ_max_ = 28.0°, θ_min_ = 1.7°
Absorption correction: integration (*SADABS*; Bruker, 1999)	*h* = −31→31
*T*_min_ = 0.188, *T*_max_ = 0.641	*k* = −12→12
38471 measured reflections	*l* = −26→26

### Refinement

**Table:**

Refinement on *F*^2^	Primary atom site location: structure-invariant direct methods
Least-squares matrix: full	Secondary atom site location: difference Fourier map
*R*[*F*^2^ > 2σ(*F*^2^)] = 0.022	Hydrogen site location: inferred from neighbouring sites
*wR*(*F*^2^) = 0.059	H-atom parameters constrained
*S* = 1.05	*w* = 1/[σ^2^(*F*_o_^2^) + (0.0337*P*)^2^ + 7.339*P*] where *P* = (*F*_o_^2^ + 2*F*_c_^2^)/3
5253 reflections	(Δ/σ)_max_ = 0.011
244 parameters	Δρ_max_ = 1.74 e Å^−^^3^
0 restraints	Δρ_min_ = −0.64 e Å^−^^3^
0 constraints	

### Fractional atomic coordinates and isotropic or equivalent isotropic displacement parameters (Å^2^)

**Table:**

	*x*	*y*	*z*	*U*_iso_*/*U*_eq_	
C1	0.93455 (12)	0.1681 (3)	0.72815 (16)	0.0287 (6)	
H1A	0.9526	0.0814	0.7502	0.034*	
H1B	0.9217	0.1393	0.6824	0.034*	
C2	0.88305 (13)	0.2110 (4)	0.76481 (18)	0.0385 (8)	
H2A	0.8564	0.1286	0.7643	0.058*	
H2B	0.8644	0.2958	0.7430	0.058*	
H2C	0.8951	0.2362	0.8107	0.058*	
C11	0.91251 (13)	0.4084 (3)	0.62384 (15)	0.0247 (6)	
C12	0.88945 (13)	0.3154 (4)	0.57400 (16)	0.0335 (7)	
H12	0.9132	0.2472	0.5537	0.040*	
C13	0.83239 (14)	0.3221 (4)	0.55403 (18)	0.0415 (8)	
H13	0.8171	0.2584	0.5202	0.050*	
C14	0.79741 (14)	0.4216 (4)	0.58318 (18)	0.0365 (7)	
C15	0.81971 (14)	0.5188 (4)	0.63099 (16)	0.0336 (7)	
H15	0.7962	0.5902	0.6496	0.040*	
C16	0.87698 (13)	0.5096 (3)	0.65111 (15)	0.0287 (6)	
H16	0.8923	0.5745	0.6845	0.034*	
C17	0.70206 (17)	0.4959 (6)	0.5975 (3)	0.0676 (14)	
H17A	0.6636	0.4817	0.5772	0.101*	
H17B	0.7118	0.6003	0.5966	0.101*	
H17C	0.7038	0.4617	0.6435	0.101*	
C21	1.01949 (12)	0.2838 (3)	0.59644 (14)	0.0245 (6)	
C22	1.02334 (13)	0.1309 (3)	0.59963 (15)	0.0268 (6)	
H22	1.0068	0.0799	0.6344	0.032*	
C23	1.05087 (13)	0.0534 (3)	0.55277 (16)	0.0288 (6)	
H23	1.0540	−0.0502	0.5560	0.035*	
C24	1.07393 (13)	0.1270 (4)	0.50081 (16)	0.0294 (6)	
C25	1.07051 (14)	0.2790 (4)	0.49653 (16)	0.0341 (7)	
H25	1.0862	0.3294	0.4610	0.041*	
C26	1.04377 (13)	0.3562 (3)	0.54489 (16)	0.0295 (6)	
H26	1.0421	0.4601	0.5427	0.035*	
C27	1.1244 (3)	0.1104 (5)	0.4032 (3)	0.0730 (17)	
H27A	1.1420	0.0368	0.3759	0.109*	
H27B	1.1533	0.1806	0.4201	0.109*	
H27C	1.0947	0.1623	0.3763	0.109*	
C44	0.7033 (5)	−0.0784 (7)	0.5960 (4)	0.128 (4)	
H44A	0.7209	−0.0644	0.5534	0.154*	
H44B	0.6678	−0.1352	0.5877	0.154*	
O3	0.74324 (16)	−0.1558 (4)	0.6450 (2)	0.0791 (10)	
C41	0.75325 (17)	−0.0776 (5)	0.7055 (2)	0.0461 (9)	
H41A	0.7941	−0.0731	0.7198	0.055*	
H41B	0.7321	−0.1202	0.7415	0.055*	
C42	0.7289 (4)	0.0785 (8)	0.6826 (4)	0.115 (3)	
H42A	0.7078	0.1229	0.7183	0.138*	
H42B	0.7603	0.1450	0.6729	0.138*	
N	0.97751 (9)	0.2854 (3)	0.72518 (11)	0.0227 (5)	
P	0.98471 (3)	0.38920 (7)	0.65760 (4)	0.02138 (14)	
Cl	1.08170 (4)	0.80680 (9)	0.70523 (5)	0.0473 (2)	
Au	1.029954 (5)	0.597871 (11)	0.683068 (5)	0.02524 (5)	
O1	0.74131 (11)	0.4147 (3)	0.56141 (16)	0.0535 (8)	
O2	1.10005 (11)	0.0398 (3)	0.45758 (13)	0.0411 (6)	
C43	0.6922 (5)	0.0576 (11)	0.6251 (5)	0.165 (5)	
H43A	0.6523	0.0608	0.6370	0.198*	
H43B	0.6979	0.1374	0.5930	0.198*	

### Atomic displacement parameters (Å^2^)

**Table:**

	*U*^11^	*U*^22^	*U*^33^	*U*^12^	*U*^13^	*U*^23^
C1	0.0257 (14)	0.0246 (14)	0.0351 (16)	−0.0070 (11)	−0.0025 (12)	0.0033 (12)
C2	0.0240 (14)	0.0454 (19)	0.0458 (19)	−0.0060 (14)	0.0009 (13)	0.0105 (16)
C11	0.0255 (13)	0.0239 (14)	0.0241 (13)	0.0025 (11)	−0.0019 (11)	0.0018 (11)
C12	0.0314 (15)	0.0355 (17)	0.0331 (16)	0.0056 (13)	−0.0025 (13)	−0.0108 (14)
C13	0.0344 (17)	0.047 (2)	0.0407 (19)	0.0064 (15)	−0.0116 (14)	−0.0181 (16)
C14	0.0280 (15)	0.0442 (19)	0.0360 (18)	0.0052 (14)	−0.0073 (13)	−0.0009 (14)
C15	0.0317 (16)	0.0338 (17)	0.0350 (16)	0.0087 (13)	−0.0009 (13)	−0.0030 (14)
C16	0.0304 (15)	0.0286 (15)	0.0262 (14)	0.0046 (12)	−0.0028 (12)	−0.0043 (12)
C17	0.0301 (19)	0.080 (3)	0.091 (4)	0.018 (2)	−0.009 (2)	−0.020 (3)
C21	0.0239 (13)	0.0236 (14)	0.0256 (14)	0.0011 (11)	−0.0011 (11)	−0.0025 (11)
C22	0.0289 (15)	0.0243 (14)	0.0273 (15)	−0.0001 (11)	0.0017 (12)	0.0004 (11)
C23	0.0283 (15)	0.0234 (14)	0.0344 (16)	0.0030 (12)	−0.0003 (12)	−0.0045 (12)
C24	0.0251 (14)	0.0310 (15)	0.0324 (16)	0.0009 (12)	0.0033 (12)	−0.0081 (13)
C25	0.0397 (17)	0.0331 (17)	0.0304 (16)	−0.0013 (14)	0.0099 (13)	−0.0006 (13)
C26	0.0338 (16)	0.0249 (14)	0.0301 (16)	0.0027 (12)	0.0051 (13)	0.0019 (12)
C27	0.097 (4)	0.057 (3)	0.073 (3)	−0.025 (3)	0.058 (3)	−0.029 (2)
C44	0.237 (11)	0.060 (4)	0.077 (4)	0.002 (5)	−0.059 (6)	0.001 (3)
O3	0.071 (2)	0.072 (2)	0.094 (3)	0.0068 (19)	−0.001 (2)	−0.015 (2)
C41	0.0394 (19)	0.057 (2)	0.042 (2)	−0.0079 (17)	0.0039 (16)	−0.0123 (18)
C42	0.172 (9)	0.085 (5)	0.087 (5)	0.024 (5)	0.011 (5)	−0.013 (4)
N	0.0215 (11)	0.0234 (11)	0.0228 (11)	−0.0029 (9)	−0.0022 (9)	0.0016 (9)
P	0.0227 (3)	0.0190 (3)	0.0222 (3)	0.0010 (3)	0.0003 (3)	−0.0005 (3)
Cl	0.0596 (6)	0.0271 (4)	0.0557 (5)	−0.0170 (4)	0.0083 (4)	−0.0046 (4)
Au	0.03037 (7)	0.01883 (7)	0.02676 (7)	−0.00279 (4)	0.00365 (4)	0.00018 (4)
O1	0.0284 (12)	0.069 (2)	0.0603 (18)	0.0115 (12)	−0.0135 (12)	−0.0204 (14)
O2	0.0434 (14)	0.0348 (13)	0.0473 (14)	−0.0030 (11)	0.0187 (11)	−0.0139 (11)
C43	0.239 (12)	0.156 (8)	0.101 (6)	0.127 (9)	0.019 (7)	0.032 (6)

### Geometric parameters (Å, °)

**Table:**

C1—N	1.480 (4)	C23—H23	0.9500
C1—C2	1.520 (5)	C24—O2	1.362 (4)
C1—H1A	0.9900	C24—C25	1.392 (5)
C1—H1B	0.9900	C25—C26	1.393 (4)
C2—H2A	0.9800	C25—H25	0.9500
C2—H2B	0.9800	C26—H26	0.9500
C2—H2C	0.9800	C27—O2	1.430 (5)
C11—C16	1.389 (4)	C27—H27A	0.9800
C11—C12	1.399 (4)	C27—H27B	0.9800
C11—P	1.802 (3)	C27—H27C	0.9800
C12—C13	1.382 (4)	C44—C43	1.406 (10)
C12—H12	0.9500	C44—O3	1.498 (8)
C13—C14	1.387 (5)	C44—H44A	0.9900
C13—H13	0.9500	C44—H44B	0.9900
C14—O1	1.369 (4)	O3—C41	1.423 (5)
C14—C15	1.389 (5)	C41—C42	1.594 (8)
C15—C16	1.389 (4)	C41—H41A	0.9900
C15—H15	0.9500	C41—H41B	0.9900
C16—H16	0.9500	C42—C43	1.415 (12)
C17—O1	1.427 (5)	C42—H42A	0.9900
C17—H17A	0.9800	C42—H42B	0.9900
C17—H17B	0.9800	N—N^i^	1.411 (4)
C17—H17C	0.9800	N—P	1.681 (2)
C21—C26	1.392 (4)	P—Au	2.2269 (7)
C21—C22	1.400 (4)	Cl—Au	2.2927 (8)
C21—P	1.809 (3)	Au—Au^i^	3.1414 (2)
C22—C23	1.381 (4)	C43—H43A	0.9900
C22—H22	0.9500	C43—H43B	0.9900
C23—C24	1.390 (5)		
			
N—C1—C2	114.1 (3)	C26—C25—H25	120.4
N—C1—H1A	108.7	C21—C26—C25	121.1 (3)
C2—C1—H1A	108.7	C21—C26—H26	119.4
N—C1—H1B	108.7	C25—C26—H26	119.4
C2—C1—H1B	108.7	O2—C27—H27A	109.5
H1A—C1—H1B	107.6	O2—C27—H27B	109.5
C1—C2—H2A	109.5	H27A—C27—H27B	109.5
C1—C2—H2B	109.5	O2—C27—H27C	109.5
H2A—C2—H2B	109.5	H27A—C27—H27C	109.5
C1—C2—H2C	109.5	H27B—C27—H27C	109.5
H2A—C2—H2C	109.5	C43—C44—O3	105.4 (6)
H2B—C2—H2C	109.5	C43—C44—H44A	110.7
C16—C11—C12	118.3 (3)	O3—C44—H44A	110.7
C16—C11—P	119.8 (2)	C43—C44—H44B	110.7
C12—C11—P	121.7 (2)	O3—C44—H44B	110.7
C13—C12—C11	120.5 (3)	H44A—C44—H44B	108.8
C13—C12—H12	119.8	C41—O3—C44	113.2 (4)
C11—C12—H12	119.8	O3—C41—C42	99.3 (4)
C12—C13—C14	120.2 (3)	O3—C41—H41A	111.9
C12—C13—H13	119.9	C42—C41—H41A	111.9
C14—C13—H13	119.9	O3—C41—H41B	111.9
O1—C14—C13	115.2 (3)	C42—C41—H41B	111.9
O1—C14—C15	124.4 (3)	H41A—C41—H41B	109.6
C13—C14—C15	120.4 (3)	C43—C42—C41	107.9 (6)
C16—C15—C14	118.7 (3)	C43—C42—H42A	110.1
C16—C15—H15	120.6	C41—C42—H42A	110.1
C14—C15—H15	120.6	C43—C42—H42B	110.1
C11—C16—C15	121.9 (3)	C41—C42—H42B	110.1
C11—C16—H16	119.1	H42A—C42—H42B	108.4
C15—C16—H16	119.1	N^i^—N—C1	117.2 (2)
O1—C17—H17A	109.5	N^i^—N—P	117.71 (19)
O1—C17—H17B	109.5	C1—N—P	123.29 (19)
H17A—C17—H17B	109.5	N—P—C11	102.49 (13)
O1—C17—H17C	109.5	N—P—C21	109.45 (13)
H17A—C17—H17C	109.5	C11—P—C21	104.82 (13)
H17B—C17—H17C	109.5	N—P—Au	111.57 (9)
C26—C21—C22	118.6 (3)	C11—P—Au	115.57 (9)
C26—C21—P	119.4 (2)	C21—P—Au	112.28 (10)
C22—C21—P	122.0 (2)	P—Au—Cl	175.98 (3)
C23—C22—C21	120.8 (3)	P—Au—Au^i^	87.787 (19)
C23—C22—H22	119.6	Cl—Au—Au^i^	95.61 (3)
C21—C22—H22	119.6	C14—O1—C17	117.4 (3)
C22—C23—C24	119.9 (3)	C24—O2—C27	117.1 (3)
C22—C23—H23	120.0	C44—C43—C42	110.0 (7)
C24—C23—H23	120.0	C44—C43—H43A	109.7
O2—C24—C23	114.9 (3)	C42—C43—H43A	109.7
O2—C24—C25	124.7 (3)	C44—C43—H43B	109.7
C23—C24—C25	120.3 (3)	C42—C43—H43B	109.7
C24—C25—C26	119.2 (3)	H43A—C43—H43B	108.2
C24—C25—H25	120.4		
			
C16—C11—C12—C13	−1.8 (5)	N^i^—N—P—C11	−160.78 (18)
P—C11—C12—C13	173.0 (3)	C1—N—P—C11	35.1 (3)
C11—C12—C13—C14	0.2 (6)	N^i^—N—P—C21	88.37 (19)
C12—C13—C14—O1	−177.5 (4)	C1—N—P—C21	−75.8 (2)
C12—C13—C14—C15	2.3 (6)	N^i^—N—P—Au	−36.5 (2)
O1—C14—C15—C16	176.8 (3)	C1—N—P—Au	159.4 (2)
C13—C14—C15—C16	−3.0 (5)	C16—C11—P—N	79.6 (3)
C12—C11—C16—C15	1.1 (5)	C12—C11—P—N	−95.1 (3)
P—C11—C16—C15	−173.8 (3)	C16—C11—P—C21	−166.2 (2)
C14—C15—C16—C11	1.3 (5)	C12—C11—P—C21	19.2 (3)
C26—C21—C22—C23	0.3 (5)	C16—C11—P—Au	−42.0 (3)
P—C21—C22—C23	−179.1 (2)	C12—C11—P—Au	143.3 (2)
C21—C22—C23—C24	−1.5 (5)	C26—C21—P—N	−162.5 (2)
C22—C23—C24—O2	180.0 (3)	C22—C21—P—N	16.8 (3)
C22—C23—C24—C25	1.4 (5)	C26—C21—P—C11	88.2 (3)
O2—C24—C25—C26	−178.4 (3)	C22—C21—P—C11	−92.5 (3)
C23—C24—C25—C26	0.0 (5)	C26—C21—P—Au	−38.0 (3)
C22—C21—C26—C25	1.2 (5)	C22—C21—P—Au	141.3 (2)
P—C21—C26—C25	−179.5 (2)	C13—C14—O1—C17	169.4 (4)
C24—C25—C26—C21	−1.3 (5)	C15—C14—O1—C17	−10.4 (6)
C43—C44—O3—C41	5.1 (10)	C23—C24—O2—C27	179.9 (4)
C44—O3—C41—C42	−14.9 (7)	C25—C24—O2—C27	−1.5 (6)
O3—C41—C42—C43	20.2 (8)	O3—C44—C43—C42	9.3 (13)
C2—C1—N—N^i^	93.9 (3)	C41—C42—C43—C44	−18.8 (12)
C2—C1—N—P	−101.9 (3)		

Symmetry codes: (i) −*x*+2, *y*, −*z*+3/2.

### Hydrogen-bond geometry (Å, °)

**Table:**

*D*—H···*A*	*D*—H	H···*A*	*D*···*A*	*D*—H···*A*
C1—H1A···Cl^ii^	0.99	2.80	3.592 (3)	137.
C13—H13···O1^iii^	0.95	2.61	3.549 (4)	170.
C15—H15···O3^iv^	0.95	2.63	3.497 (5)	152.
C17—H17B···O3^iv^	0.98	2.53	3.444 (7)	156.

Symmetry codes: (ii) −*x*+2, *y*−1, −*z*+3/2; (iii) −*x*+3/2, −*y*+1/2, −*z*+1; (iv) *x*, *y*+1, *z*.
